# Adhesion Molecule Profile and the Effect of Anti-VLA-4 mAb Treatment in Experimental Autoimmune Encephalomyelitis, a Mouse Model of Multiple Sclerosis

**DOI:** 10.3390/ijms23094637

**Published:** 2022-04-22

**Authors:** Grażyna Pyka-Fościak, Grzegorz J. Lis, Jan A. Litwin

**Affiliations:** Department of Histology, Jagiellonian University Medical College, 31-034 Kraków, Poland; grzegorz.lis@uj.edu.pl (G.J.L.); j.a.litwin@uj.edu.pl (J.A.L.)

**Keywords:** experimental autoimmune encephalomyelitis (EAE), anti-VLA-4 mAb, inflammation, adhesion molecules, multiple sclerosis

## Abstract

In the course of multiple sclerosis (MS) and its animal model, experimental autoimmune encephalomyelitis (EAE), the infiltration of lymphocytes and other inflammatory cells across the blood–brain barrier is associated with interactions between adhesion molecules expressed by infiltrating cells and vascular endothelium. Monoclonal antibodies (mAb) against the α4 subunit of α4-β1 integrin (VLA-4) show beneficial effects in both MS and EAE. (1) Background: The aim of this study was to examine the expression of selected adhesion molecules: VLA-4, VCAM-1, LFA-1, ICAM-1 and PECAM-1 in the successive phases of EAE and the effect of anti-VLA-4 mAb treatment on that expression. (2) Methods: EAE was induced in C57BL/6 mice by immunization with MOG_35–55_ peptide. The animals were killed in three successive phases of the disease: onset (day 13), peak (day 18) and chronic (day 28). Frozen sections of the lumbar spinal cord were examined by quantitative immunofluorescence microscopy. The expression of the studied molecules was quantified as the percentage of the cross-sectioned spinal cord lesion area occupied by immunopositive structures. (3) Results: The expression of the studied molecules showed two temporal patterns: (1) an increase in the onset phase, a maximum in the peak phase and a decrease in the chronic phase, which corresponded to the temporal pattern of the clinical score, the number of lesions and the inflammation level (ICAM-1, LFA-1 and PECAM-1), and (2) an increase in the peak phase and no significant change or further increase in the chronic phase (VCAM-1, VLA-4). Among the molecules studied, ICAM-1 and LFA-1 exhibited the highest expression levels in the peak phase of EAE. Anti-VLA-4 mAb inhibited the expression of not only VLA-4 but also other adhesion molecules. (4) Conclusions: The interactions of adhesion molecules governing the migration of leukocytes across the blood–brain barrier change in the successive phases of EAE. The therapeutic mechanism of anti-VLA-4 mAb treatment seems to include a complex influence on a variety of adhesion molecules expressed by infiltrating cells and vascular endothelium.

## 1. Introduction

The specific expression of adhesion molecules at the blood–brain barrier (BBB) level is a pathogenic symptom in neuroinflammatory diseases such as multiple sclerosis (MS) and its animal model, experimental autoimmune encephalomyelitis (EAE) [1,2,3]. When autoreactive pathogenic Th cells break down the BBB, complex molecular interactions occur between adhesion molecules in the cell membranes of the infiltrating cells and capillary endothelial cells [3,4]. The invasion of Th cells into the brain and spinal cord leads to the formation of lesions exhibiting an inflammatory response, demyelination, gliosis (scarring), axonal injury and axonal loss.

Studies on adhesion molecules and their expression patterns at the BBB during leukocyte infiltration in the course of EAE have revealed that these molecules are a valuable target for the evaluation of therapeutic interventions at the BBB. The interactions between α4-β1 integrin (VLA-4), lymphocyte function-associated antigen (LFA-1), vascular cell adhesion molecule (VCAM-1) and intercellular adhesion molecule (ICAM-1) influence the adhesion and migration of infiltrating cells [5,6]. Another important adhesion molecule involved in the transendothelial migration of leukocytes is platelet-endothelial cell adhesion molecule-1 (PECAM-1, CD31) [7]. It shows homophilic interaction through its extracellular domain (sPECAM-1) and also binds to integrin αv-β3, an adhesion molecule found on endothelial cells [8]. PECAM-1 stabilizes BBB permeability and regulates vascular integrity and immune cell trafficking [7]. 

Blocking of adhesion molecules is an effective strategy to prevent CNS inflammation in MS and its animal model, EAE [4,9]. Natalizumab, used in the therapy of MS, is a monoclonal antibody (mAb) against the α4 subunit of α4-β1 (VLA-4) and α4-β7 integrins; its main mechanism of action is to block the binding of lymphocyte α4 integrins to their endothelial receptors (VCAM-1), preventing inflammatory cells from crossing the BBB, entering the CNS and attacking myelin sheaths [10,11]. The results of studies on the effect of natalizumab (anti-VLA-4) therapy in EAE are partly confusing. Anti-VLA-4 mAb treatment was found to suppress inflammatory infiltration and clinical scores during the progression of EAE [10,12]. Preclinical administration of anti-VLA-4 mAb delayed the onset and reduced the severity of the disease [12,13], but the treatment at the peak of acute disease or during remission exacerbated disease relapses [13]. Administration of synthetic VLA-4 antagonist during the chronic phase of EAE had essentially no effect; moreover, disease worsening occurred after cessation of treatment. The lack of effect after late anti-VLA-4 treatment can be associated with changes in the expression of adhesion molecules at different stages of the disease development [14].

The variable activity of inflammatory infiltrates in the CNS, characteristic of MS and EAE, probably reflects the differentiated expression of adhesion molecules associated with leukocyte migration. In this context, we used quantitative immunofluorescence on EAE spinal cord sections to investigate the expression of VCAM-1/VLA-4 and ICAM-1/LFA-1 pairs of interacting adhesion molecules, and we compared it with the expression of PECAM-1 and studied the effect of anti-VLA-4 mAb treatment on that expression in the successive phases of EAE. The spinal cord was chosen because it is the dominant location of EAE lesions [15], and sometimes EAE affects the spinal cord but not the brain [16].

## 2. Results

**Figure 1:**
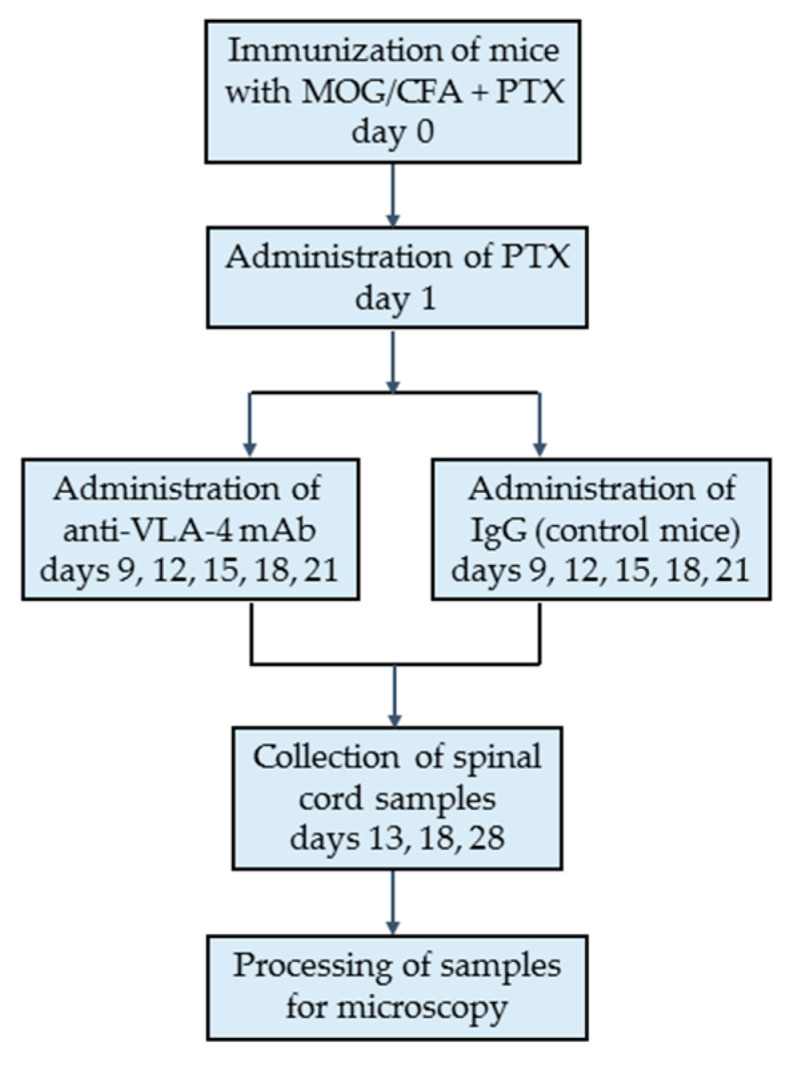
Outline of the study.

The outline of the study is presented in Figure 1.

### 2.1. Effect of Anti-VLA-4 mAb and IgG Treatment on the Development of Progressive EAE

**Figure 2:**
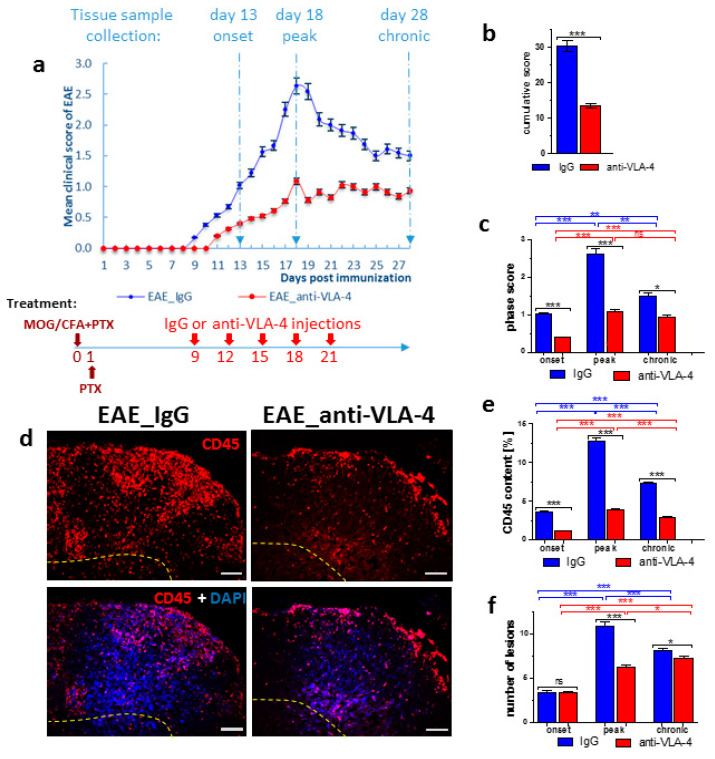
EAE mice were immunized with MOG and CFA (day 0) and PTx (day 0 and 1) (**a**, brown arrows). The clinical scores of EAE were assessed in experiments in which immunized mice were treated with IgG (**a**, blue points) or anti-VLA-4 mAb (**a**, red points) every third day for 12 days (**a**, red arrows). Days of tissue collection and disease phases are indicated (**a**, upper part). Histograms show the cumulative clinical score (means ± SEM) for IgG and anti-VLA-4 mice (**b**) and mean score in onset, peak and chronic phases of EAE (**c**). CD45 immunostaining of spinal cord sections shows inflammatory infiltration in the peak phase of EAE for IgG and anti-VLA-4 groups (in all micrographs, yellow dashed line marks the border between white and gray matter) (**d**). In order to assess inflammation degree, the area occupied by CD45 immunopositive cells was quantified as percentage of cross-sectioned cord surface area (**e**). Anti-VLA-4 treatment attenuated the disease clinical scores (**a**–**c**), the area of inflammatory lesions compared to IgG treatment (**e**) and the number of lesions (**f**). Data are presented as means ± SEM; *n* = 5 per group. nf—not found. Statistical significance was verified using a two-sided Mann–Whitney test at 0.05 confidence level (*** *p* < 0.001; ** *p* < 0.01; * *p* < 0.05; ns—not significant). Magnification is indicated by scale bars (=100 µm).

The clinical course of EAE mice was attenuated by anti-VLA-4 mAb and IgG treatment. Anti-VLA-4 mAb treatment significantly delayed the onset in immunized mice and reduced the severity of clinical EAE compared to the IgG group (Figure 2a). The experimental timeline is diagramed in Figure 2a.

On the basis of the protocols included in the Hooke Kits™ EAE Emulsion (Hooke Laboratories, Lawrence, MA, USA), the three phases of EAE were characterized by the following mean clinical scores: onset: 1.02 ± 0.05 for IgG group vs. 0.4 ± 0.02 for anti-VLA-4 group; peak: 2.64 ± 0.13 for IgG group vs. 1.09 ± 0.05 for anti-VLA-4 group; chronic: 1.5 ± 0.08 for IgG group vs. 0.94 ± 0.05 for anti-VLA-4 group (Figure 2a,c). In the chronic phase, the clinical scores decreased in the IgG group, but they remained at the same level in the anti-VLA-treated mice. The cumulative EAE score was significantly higher in the IgG group than in anti-VLA-4 mice (30.31 ± 1.49 and 13.53 ± 0.66, respectively; Figure 2b). 

### 2.2. CD45 Expression

CD45, also known as leukocyte common antigen (LCA), expressed by the vast majority of hematolymphoid lineage cells, is widely used for the detection of leukocytes. Hence, a CD45 antibody was employed in this study to check if anti-VLA-4 mAb treatment affected inflammatory infiltrates, and the immunostained area was quantified by a morphometric analysis (Figure 2d,e). Immunoreactivity for CD45 showed large accumulations of leukocytes, mainly in the white matter of the spinal cords of the immunized mice. In all phases of the disease, leukocytes formed local aggregates with high cell densities, indicative of the presence of inflammatory foci. The degree of inflammation, expressed as the percentage of the immunopositive section surface area (Figure 2e) in both anti-VLA-4 and IgG groups, increased from the onset to the peak phase and decreased in the chronic phase; however, the values in the latter phase were higher than those in the onset phase, indicating that inflammation did not fully retreat. In all EAE phases, the values were significantly lower in anti-VLA-4-treated mice compared to IgG-treated mice (onset phase: 1.09 ± 0.06% vs. 3.58 ± 0.09%; peak phase: 3.85 ± 0.15% vs. 12.8 ± 0.36%; chronic phase: 2.82 ± 0.15% vs. 7.3 ± 0.15%). 

CD45 immunostaining allowed for assessing the number of inflammatory lesions per group and phase. In IgG-treated mice, during the successive phases, it increased from 3.42 ± 0.18 to 10.92 ± 0.45 and then slightly decreased to 8.15 ± 0.22. Anti-VLA-4 treatment had no effect in the onset phase, reduced the number of lesions by nearly a half in the peak phase and caused further, although weak, reduction in the chronic phase (Figure 2f).

### 2.3. ICAM-1/LFA-1 Expression

**Figure 3:**
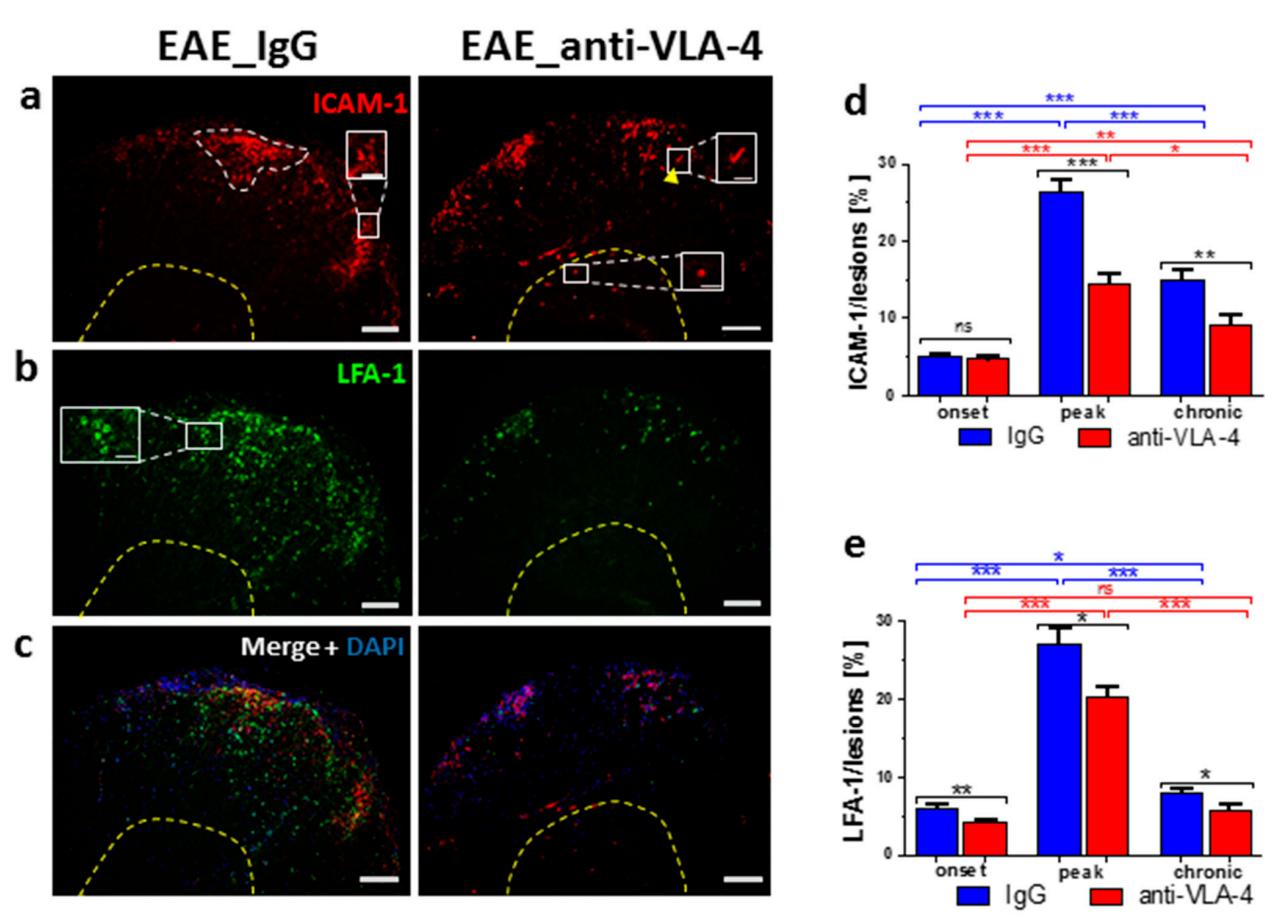
Immunostaining of ICAM-1 (**a**), LFA-1 (**b**) and their overlapping fluorescence with DAPI nuclear staining (**c**) in cross-sectioned spinal cords of IgG- and anti-VLA-4-treated mice in the peak phase of EAE, as well as their quantitative measurements. ICAM-1 and LFA-1 expression was quantified as percentage of lesion area occupied by immunopositive structures (**d**,**e**); white dotted line marks an exemplary lesion in which expression of adhesion molecules was measured. Localization of LFA-1 is associated with inflammatory cells (**b,c**), and localization of ICAM-1 is associated with inflammatory cells (**a**, inset), a few nerve cells in gray matter (**a**, inset) and capillaries (**a**, inset, arrowhead). Data are presented as means ± SEM; *n* = 5 per group. Statistical significance was verified using a two-sided Mann–Whitney test at 0.05 confidence level (*** *p* < 0.001; ** *p* < 0.01; * *p* < 0.05; ns—not significant). Magnification is indicated by scale bars ((**a**–**c**) = 100 µm; insets = 25 µm).

Generally, anti-VLA-4 mAb treatment lowered both ICAM-1 and LFA-1 expression in inflammatory lesions. This effect was absent only in the case of ICAM-1 in the onset phase (Figure 3d). During the progression of EAE in IgG-treated mice, ICAM-1 expression increased from 5.04 ± 0.39% to 26.27 ± 1.67% and then dropped to 14.92 ± 1.18%. The respective values for anti-VLA-4-treated mice were 4.68 ± 0.32%, 14.42 ± 1.4% and 9.2 ± 1.11%. ICAM-1 was expressed by inflammatory cells, capillaries and occasional nerve-like cells in gray matter (Figure 3a,c).

A similar pattern, i.e., a rise from onset to peak (from 6.04 ± 0.52% to 27.01 ± 2.11%) and a decrease in the chronic phase (to 7.97 ± 0.47%), was observed in spinal cords immunostained for LFA-1. Anti-VLA-4 mAb treatment slightly but significantly inhibited LFA-1 expression in all phases of EAE (Figure 3e). LFA-1 immunopositive inflammatory cells were located exclusively in the white matter (Figure 3b,c).

### 2.4. VCAM-1/VLA-4 Expression

**Figure 4:**
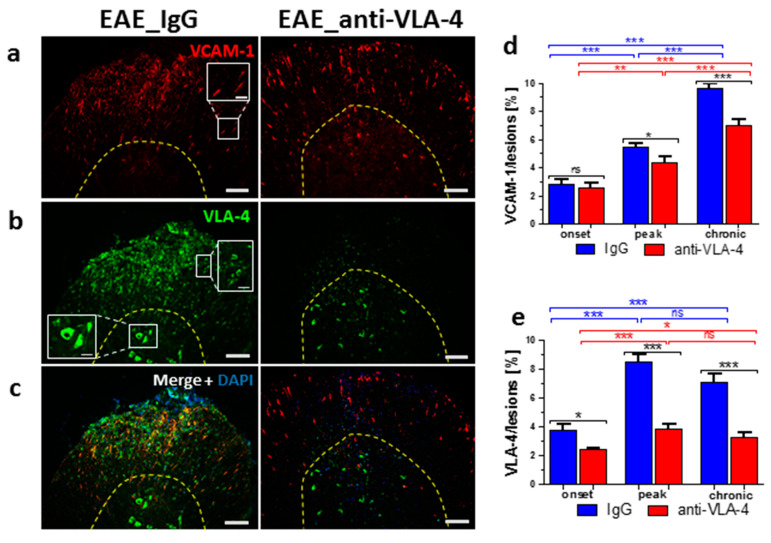
Immunostaining of VCAM-1 (**a**), VLA-4 (**b**) and overlapping fluorescence with DAPI (**c**) in cross-sectioned spinal cords of IgG- and anti-VLA-4-treated mice in the peak phase of EAE, as well as their quantitative measurements. VCAM-1 and VLA-4 expression was quantified as percentage of lesion area occupied by immunofluorescent structures (**d**,**e**). VCAM-1 is mainly localized in capillaries (**a**, inset), and localization of VLA-4 is associated with inflammatory cells in white matter (**b**, inset) and in some neuron-like cells in gray matter (**b**, inset). Data are presented as means ± SEM; *n* = 5 per group; nf—not found. Statistical significance was verified using a two-sided Mann–Whitney test at 0.05 confidence level (*** *p* < 0.001; ** *p* < 0.01; * *p* < 0.05; ns—not significant). Magnification is indicated by scale bars ((**a**–**c**) = 100 µm; insets = 25 µm).

In IgG-treated mice, the expression of VCAM-1, mostly manifested in capillary endothelia (Figure 4a,c), gradually increased in the successive phases (from 2.83 ± 0.34% to 5.46 ± 0.29% and 9.6 ± 0.35%, respectively). Anti-VLA-4 mAb treatment was ineffective in the onset phase but significantly lowered VCAM-1 expression only in the next two phases (to 4.37 ± 0.44% and 7.0 ± 0.42, respectively; Figure 4d).

VLA-4 expressed in inflammatory cells in the white matter and in some neuron-like cells of the gray matter (Figure 4b, inset) in IgG-treated animals showed an increase from 3.78 ± 0.39% in the onset phase to 8.47 ± 0.49% in the peak phase and remained at a comparable level (7.1 ± 0.6%) in the chronic phase (Figure 4e). Anti-VLA-4 mAb treatment reduced VLA-4 expression in all phases of EAE (to 2.43 ± 0.11%, 3.86 ± 0.28% and 3.3 ± 0.32%, Figure 4e). The treatment completely abolished VLA-4 immunofluorescence in inflammatory cells, whereas neuron-like cells remained immunopositive (Figure 4b,c).

### 2.5. PECAM-1 Expression

**Figure 5:**
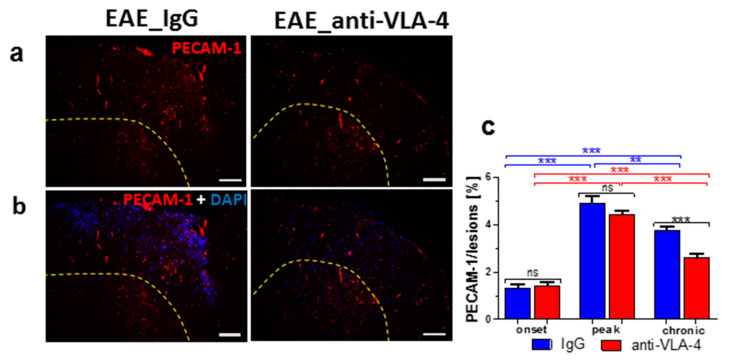
Immunostaining of PECAM-1 (**a**) and its overlapping fluorescence with DAPI (**b**) in cross-sectioned spinal cords of IgG- and anti-VLA-4-treated mice in the peak phase of EAE, as well as its quantitative measurements. PECAM-1 localization is associated with scarce cells and capillaries. Percentage values in (**c**) concern lesion area occupied by immunopositive structures. Data are presented as means ± SEM; *n* = 5 per group. Statistical significance was verified using a two-sided Mann–Whitney test at 0.05 confidence level (*** *p* < 0.001; ** *p* < 0.01; ns—not significant). Magnification is indicated by scale bars (=100 µm).

The expression of PECAM-1 was detected in some infiltrating inflammatory cells and capillary vessels (Figure 5a,b). With EAE progression, the expression of PECAM-1 significantly increased from 1.33 ± 0.15% in the onset phase to 4.91 ± 0.28% in the peak phase and decreased to 3.77 ± 0.17% in the chronic phase. Anti-VLA-4 mAb treatment significantly reduced PECAM-1 expression only in the chronic phase (to 2.59 ± 0.19%, Figure 5c).

**Figure 6:**
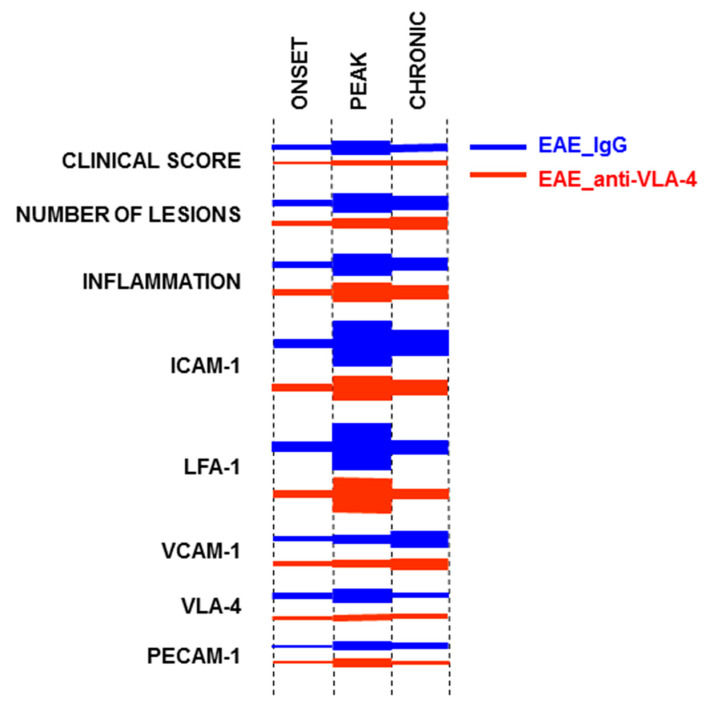
Graphic summary of intensity/expression levels and changes in the studied EAE parameters in the successive phases of the disease. Thickness of segments represents quantitated levels.

The summarized results of the study are graphically presented in Figure 6.

## 3. Discussion

The expression of adhesion molecules at the brain–blood barrier (BBB) is of pathogenic relevance in inflammatory diseases of the CNS such as MS and its animal model, EAE. The upregulation of various adhesion molecules in the brain and spinal cord tissues of MS/EAE has been demonstrated in several studies [2,3,6]. Moreover, blocking of adhesion molecules was shown to be an effective strategy to ameliorate the severity of EAE [9,10]. The inhibition of VLA-4 integrin-mediated binding of leukocytes crossing the BBB to their endothelial receptors is believed to be the basis of the therapeutic potential of natalizumab, a monoclonal antibody against alpha4 beta1integrin (VLA-4), in reducing the inflammatory and clinical symptoms of MS and EAE. A similar effect on T-cell adhesion and infiltration was observed after treatment with an antibody against ICAM-1 [17].

In the present study, we used an EAE model in which the progression of clinical scores allows for characterizing three successive phases of the disease: onset, peak and chronic (recovery). The inflammation level was assessed by measuring the expression of CD45, a general marker of proinflammatory leukocytes, which are predominant in the inflammatory foci. These cells also have a regulatory function, contributing to the development and intensity of inflammation [18].

In IgG-treated mice (treatment control), inflammatory infiltration in the spinal cord increased during the first two phases of EAE and decreased, but still remained at an elevated level, in the chronic phase. Such a pattern was also observed in our previous studies [12,19]. Anti-VLA-4 mAb treatment significantly suppressed inflammatory infiltration compared to the IgG group, delayed the clinical disease onset and reduced clinical scores in the subsequent phases. 

Quantitative analysis of the temporal expression patterns of adhesion molecules in MS and EAE can provide valuable information because many new and innovative therapeutic strategies in MS are aimed at preventing the penetration of inflammatory cells across the BBB, and this process depends on interactions between adhesion molecules. VLA-4/VCAM-1, LFA-1/ICAM-1 and PECAM-1/PECAM-1 interactions play an important role in the adhesion and migration of leukocytes across the endothelial barrier [20,21]. The lowered expression of adhesion molecules in brain endothelial cells can lead to reduced inflammatory infiltration and the inhibition of new lesion formation in EAE [6].

In order to quantify the expression of the studied adhesion molecules, we could not assess their overall expression in the entire spinal cord sections, as we did in our previous studies [12,19], because the result would depend on the number and area of lesions characterized by high inflammatory cell density, and these parameters differ between sections. Hence, we decided to measure the expression of adhesion molecules in lesions only, which show comparable inflammatory cell density.

A predominant temporal expression pattern emerged in control, IgG-treated mice: an increase in the onset phase, a maximum in the peak phase and a decrease in the chronic phase (ICAM-1/LFA-1 and PECAM-1)—this pattern corresponded to that of the clinical score, the number of lesions and the inflammation level (CD45). The pair VCAM-1/VLA-1 did not show a decrease in expression in the chronic phase—instead, an increase (VCAM-1) or no change compared with the peak phase (VLA-1) was observed.

The expression of adhesion molecules in the course of EAE has been the subject of several studies [3,22,23,24,25,26,27]. It is, however, difficult to compare the results of these studies with ours because the authors used different EAE models in mice and rats, with different methods to induce the disease, and EAE phases were not specified in a standardized manner. Generally, the reported successive changes in adhesion molecule expression corresponded to the predominant pattern described above. The maintenance of high expression levels of some adhesion molecules in the chronic phase of EAE is a novel observation indicating the differentiated involvement of adhesion molecules in the course of the disease.

In the present study, ICAM-1 and LFA-1 were found to be the dominant adhesion molecules in the peak phase of EAE, as far as the quantitative expression of the protein was concerned. Although VLA-4 upregulation in T cells is crucial for their migration into the CNS [28], lymphocytes have been reported to adhere to activated endothelium via VLA-4 and LFA-1, but their subsequent migration appears to be regulated mainly by ICAM-1 and partly by PECAM-1 [17,19]. VLA-4 immunostaining of neuron-like cells observed in the present study was surprising because, in the literature, it was reported only in some retinal and peripheral neurons [29]. We suppose that this staining resulted from some non-specific cross-reaction, especially since it was not abolished by anti-VLA-4 treatment.

Low ICAM-1 expression in the onset phase and its high levels in the peak and chronic phases seem to contradict the opinion that this adhesion molecule is involved mainly in the early phase of EAE [22,24,25]. However, it confirms the significance of ICAM-1 in the pathogenesis of EAE. Mutant mice lacking the ICAM-1 gene showed attenuated EAE with reduced T-cell infiltration in the spinal cord [27].

Upregulated expression of PECAM-1 was observed in MS lesions and could reflect vascular repair mechanisms aimed at the restoration of BBB integrity and the inhibition of T-cell migration across the BBB [7]. The importance of PECAM expression in EAE is not clear. Earlier studies suggested that it did not influence the clinical severity of the disease [26]. However, PECAM-1-deficient mice showed earlier inflammatory infiltration of the CNS and higher permeability of CNS vessels as compared with wild-type EAE mice [23]. A prominent increase in PECAM-1 expression observed in the peak phase of EAE and a weak decrease in its level in the chronic phase seem to confirm an important role of this molecule in the development of the disease. 

The novel finding of this study is that anti-VLA-4 mAb treatment inhibits the expression of not only VLA-4 but also the other adhesion molecules studied. As expected, this inhibition in the case of VLA-4 was the strongest and not phase-dependent. Interestingly, in the case of ICAM-1, VCAM-1 and PECAM-1, the inhibitory effect of anti-VLA-4 treatment was delayed and occurred in the peak and chronic phases of EAE (ICAM-1 and VCAM-1) or even only in the chronic phase (PECAM-1). These adhesion molecules are at least partly associated with endothelial cells; hence, it seems that the endothelium responds with some delay to the blocking effect of anti-VLA-4 mAb.

Anti-VLA-4 mAb treatment attenuates proinflammatory mediators, increases the production of anti-inflammatory IL-10 [30] and also—probably indirectly—downregulates metalloproteinases as well as upregulates their inhibitors, counteracting the disintegration of the vascular basal laminae [12]. The results of the present study justify the supposition that the therapeutic mechanism of anti-VLA-4 mAb treatment also includes a complex influence on a variety of adhesion molecules expressed by infiltrating cells and vascular endothelium.

## 4. Materials and Methods

### 4.1. Animals

Pathogen-free C57BL/6 mice (female, 10–11 weeks old, weight 19–24 g) were purchased from the Center for Experimental Medicine of Bialystok Medical University, Poland (strain imported from Jackson Laboratory). Mice were housed, five per cage, in the animal house of the Jagiellonian Centre for Experimental Therapeutics (JCET), Krakow, under a 12 h light–dark cycle in temperature-controlled environment (22 ± 2 °C, 55 ± 10% humidity). Standard irradiated laboratory chow and water were available ad libitum. All experiments were conducted in compliance with the Council Directive 2010/63EU of the European Parliament and of the Council of 22 September 2010 on the protection of animals used for scientific purposes and approved by the First and the Second Local Ethics Committees in Krakow, Poland (Permissions 118/2015 and 274/2018).

### 4.2. Induction of EAE

To induce EAE, naïve mice (*n =* 30) were subcutaneously immunized with 200 μL injection of Hooke Kits™ EAE Emulsion (Hooke Laboratories, Lawrence, MA, USA) containing MOG_35–55_ peptide emulsified in Complete Freund’s Adjuvant (CFA) including 4 mg/mL heat-killed Mycobacterium tuberculosis (H37Ra). On the day of MOG_35–55_ immunization and 24 h later, the mice were also injected intraperitoneally (i.p.) with 340 µL of Bordetella pertussis pertussis toxin (PTx) dissolved in phosphate-buffered saline (PBS) (Hooke Laboratories, Lawrence, MA, USA) (Figure 1 and Figure 2a).

### 4.3. Evaluation of EAE

The mice were examined daily for clinical signs of EAE from day 0 to day 28, and the clinical symptoms of EAE were scored as reported previously [12]. In short, disease severity was evaluated using a scale ranging from 0 to 3: (0) no clinical disease; 0.5—limp tip of tail; 1—limp tail; 1.5—limp tail and hind leg inhibition; 2—limp tail and weakness of hind legs; 2.5—limp tail and dragging of hind legs; 3—limp tail and complete paralysis of hind legs. The mice were examined and scored by one person blinded to the treatment.

### 4.4. Experimental Groups

The immunized EAE mice (*n* = 30) were divided into two experimental groups: anti-VLA-4 group (*n* = 15) receiving i.p. injection of 5 mg/kg of anti-VLA-4 mAb (Natalizumab, Biogen Idec, Berkshire, UK) and IgG group (IgG control group, *n* = 15) receiving 5 mg/kg IgG (Sigma-Aldrich, St. Louis, MO, USA). The injections (every 3 days, on days 9, 12, 15, 18 and 21) were continued until the appearance of first remission symptoms (Figure 1 and Figure 2a) [17,20].

In MOG_35–55_ immunized mice, the initial symptoms of the disease were observed between 9 and 14 post-immunization days (onset phase). The maximum scores (peak phase) occurred between post-immunization days 15 and 20, and then the mice partially recovered (chronic phase). In EAE mice treated with anti-VLA-4 mAb, the onset phase began later (day 11), and the peak phase was shorter (days 15 to 18). 

For histological analysis, EAE mice (anti-VLA-4 and IgG groups) were sacrificed at three different time points representing three disease phases (*n* = 5 per group and phase): onset phase (day 13), peak phase (day 18) and chronic phase (day 28) (Figure 1 and Figure 2a). 

### 4.5. Tissue Sampling and Processing

To collect spinal cords, mice were anesthetized with ketamine/xylazine cocktail (100 mg/kg and 10 mg/kg, respectively, i.p.). Next, the animals were transcardially perfused with ice-cold PBS for 10 min, followed by 4% paraformaldehyde for the next 10 min. Spinal cords were carefully removed from the vertebral canal and postfixed in the same fixative for 4 h. After overnight incubation in 5% sucrose at 4 °C, tissue was embedded in OCT (Shandon Cryomatrix, Thermo Fisher Scientific, Rockford, IL, USA) and snap-frozen at −80 °C. The study area of the spinal cord included the lumbar part, a region commonly and rapidly affected in EAE. Serial cryosections, 10 µm thick, were cut at 100 µm intervals, collected on poly-L-lysine-coated slides and air-dried. The sections were fixed with acetone for 20 min and air-dried again.

### 4.6. Immunohistochemistry

The following primary antibodies were used: rat anti-ITGA4 (VLA-4, Thermo Fisher Scientific, Rockford, IL, USA, 1:100; cat. # MA5-70075), rat anti-CD11a (LFA-1α, Thermo Fisher Scientific, Rockford, IL, USA, 1:200; cat. # 14-0111-85), rabbit anti-VCAM-1 (Thermo Fisher Scientific, Rockford, IL, USA; 1:50; cat. # PA5-86042), rabbit anti-ICAM-1 (Thermo Fisher Scientific, Rockford, IL, USA; 1:100; cat. # PA5-79430) and rat anti-PECAM-1 (CD31, Sigma-Aldrich, St. Louis, MO, USA, 2 µg/mL, cat. # CBL 1337). Anti-CD45 primary antibody for total leukocytes (Thermo Fisher Scientific, 1:100, MA1-81247) was applied to analyze the degree of inflammatory infiltration. The secondary antibodies included goat anti-rat Alexa488-conjugated antibodies (Jackson IR, West Grove, PA, USA, 1:100; cat. # 112-545-167), Cy3-conjugated goat anti-rabbit antibodies (Jackson IR, West Grove, PA, USA; 1:300; cat. # 111-165-144), goat anti-rabbit Alexa488-conjugated antibodies (Jackson IR, West Grove, PA, USA, 1:100; cat. # 111-545-144) and Cy3-conjugated goat anti-rat antibodies (Jackson IR, West Grove, PA, USA; 1:300; cat. # 112-165-167). DAPI staining was used to detect nuclei (Thermo Fisher Scientific, Rockford, IL, USA; 1.5 ug/mL; cat. # 62248).

The spinal cord sections were preincubated for 40 min in a blocking solution: PBS containing 5% normal goat serum (Sigma-Aldrich, St. Louis, MO, USA), 0.01% sodium azide, 0.05% thimerosal, 0.1% bovine serum albumin, 0.5% Triton X-100 and 2% dry milk. They were next incubated overnight at room temperature with primary antibodies and, after a rinse in PBS, incubated for 90 min with the secondary antibodies. Then, sections were washed three times in PBS and mounted in glycerol/PBS solution.

### 4.7. Microscopy, Morphometry and Image Collection

The spinal cord sections were examined using Olympus BX50 brightfield/epifluorescence microscope (Olympus, Tokyo, Japan). All low magnification images were recorded with the use of Olympus DP71 digital CCD camera, stored as TIFF files and processed for quantitative analysis using ImageJ software (NIH, Bethesda, MD, USA).

In each image, inflammatory lesions characterized by high density of cells were marked (example in Figure 2a), and the percentage of immunopositive areas was assessed in the lesions. Only in the case of CD45, a general marker of inflammation, the percentage of immunopositive areas was estimated for the whole spinal cord cross-sectional area (Figure 2e).

A total of at least 20 sections were analyzed per experimental group (*n* = 5) and phase. 

### 4.8. Data Analysis

GraphPad Prism 5.0 software (GraphPad, La Jolla, CA, USA) was used throughout this study for statistical analyses. All values are expressed as mean ± standard error of the mean (SEM). Statistical significance of the obtained results was verified using two-sided Mann–Whitney test at a confidence level of 0.05 (*** *p* < 0.001; ** *p* < 0.01; * *p* < 0.05; ns—not significant).

## Data Availability

Not applicable.
